# Supplementary material for: Resolving the full spectrum of human genome variation using Linked-Reads
Source: Genome Res. 2019 Apr;29(4):635–45. doi: 10.1101/gr.234443.118 (PMC6442396; doi:10.1101/gr.234443.118)
Supplement: Supplemental Material [file supp_gr.234443.118_Supplemental_longranger-2.2.zip › longranger-2.2.tar.bz2!/lr2.2-public/lib/stan/cmdstan/stan/doc/api/html/math_2functions_2binomial__coefficient__log_8hpp_source.html]

Stan: src/stan/math/functions/binomial\_coefficient\_log.hpp Source File


|  |  |
| --- | --- |
|  | Stan  2.5.0  probability, sampling & optimization |


- Main Page
- Related Pages
- Namespaces
- Classes
- Files

- File List
- File Members

All Classes Namespaces Files Functions Variables Typedefs Enumerations Enumerator Friends Macros Pages

- src
- stan
- math
- functions

binomial\_coefficient\_log.hpp

Go to the documentation of this file.

1 #ifndef STAN\_\_MATH\_\_FUNCTIONS\_\_BINOMIAL\_COEFFICIENT\_LOG\_HPP

2 #define STAN\_\_MATH\_\_FUNCTIONS\_\_BINOMIAL\_COEFFICIENT\_LOG\_HPP

3

4 #include <boost/math/special\_functions/gamma.hpp>

5 #include <boost/math/tools/promotion.hpp>

6

7 namespace stan {

8

9  namespace math {

10

61  template <typename T\_N, typename T\_n>

62  inline typename boost::math::tools::promote\_args<T\_N, T\_n>::type

63  binomial\_coefficient\_log(const T\_N N, const T\_n n) {

64  using std::log;

65  using boost::math::lgamma;

66

67  const double cutoff = 1000;

68  if ((N < cutoff) || (N - n < cutoff)) {

69  return lgamma(N + 1.0) - lgamma(n + 1.0) - lgamma(N - n + 1.0);

70  } else {

71  return n \* log(N - n) + (N + 0.5) \* log(N/(N-n))

72  + 1/(12\*N) - n - 1/(12\*(N-n)) - lgamma(n + 1.0);

73  }

74  }

75

76  }

77 }

78

79 #endif

stan::math::binomial\_coefficient\_log

boost::math::tools::promote\_args< T\_N, T\_n >::type binomial\_coefficient\_log(const T\_N N, const T\_n n)

Return the log of the binomial coefficient for the specified arguments.

**Definition:** binomial\_coefficient\_log.hpp:63

stan::agrad::lgamma

fvar< T > lgamma(const fvar< T > &x)

**Definition:** lgamma.hpp:15

stan::math::lgamma

double lgamma(double x)

**Definition:** lgamma.hpp:31

stan::math::log

Eigen::Matrix< T, Rows, Cols > log(const Eigen::Matrix< T, Rows, Cols > &m)

Return the element-wise logarithm of the matrix or vector.

**Definition:** log.hpp:16

stan::agrad::log

fvar< T > log(const fvar< T > &x)

**Definition:** log.hpp:15


---

[ Stan Home Page ]

*© 2011–2014,
Stan Development Team.*
